# Sepsis profile among preterm infants with enterostomy and fecal transfer: a multicenter retrospective cohort study

**DOI:** 10.1186/s40348-026-00227-2

**Published:** 2026-04-10

**Authors:** Zeina Al-Kudsi, Christian Wieg, Mats Ingmar Fortmann, Kirstin Faust, Egbert Herting, Knud Linnemann, Ursula Weller, Jenny Potratz, Wolfgang Göpel, Alexander Humberg

**Affiliations:** 1 Department of General Pediatrics, University Hospital Münster, Albert-Schweitzer-Campus A1, Münster, 48149 Germany; 2 Department of Neonatology and Pediatric Intensive Care, Clinical Centre Aschaffenburg-Alzenau, Aschaffenburg, Germany; 3 Department of Pediatrics, University Hospital Schleswig-Holstein, Campus Lübeck, Lübeck, Germany; 4 Department of Neonatology and Pediatric Intensive Care, University Hospital Greifswald, Greifswald, Germany; 5 Department of Pediatrics, University of Bielefeld, Bielefeld, Germany

**Keywords:** Enterostomy, Mucous fistula refeeding, Preterm infants, Sepsis, Neonatal surgery, VLBWI

## Abstract

**Background:**

Very low birth weight infants (VLBWI, < 1500 g) with abdominal complications often require enterostomy, interrupting physiological intestinal passage. Mucous fistula refeeding (MFR; fecal transfer) aims to restore intestinal continuity and to improve nutrient absorption, yet concerns about infection risk persist.

In this multicenter retrospective study, we evaluated the association between MFR and clinical risk profiles in VLBWI with enterostomy until stoma reversal. VLBWI with enterostomy were treated between 2009 and 2022 in five centers of the German Neonatal Network (GNN). Data of infants were grouped by local refeeding practice (MFR vs. no MFR). Logistic regression analyses assessed infections, cholestasis, and growth outcomes, adjusted for gestational age, birth weight, central line use, probiotic use, and antibiotic exposure.

**Results:**

Of 60 infants, 26 received MFR and 34 did not. MFR infants showed lower rates of blood-culture–proven sepsis (11.5% vs. 36.4%, *p* = 0.03) and clinical sepsis (50.0% vs. 78.8%, *p* = 0.02). Recurrent infections occurred less often (46.2% vs. 75.8%, *p* = 0.02). Cholestasis and growth parameters were comparable. MFR remained independently associated with reduced odds of blood-culture–proven sepsis (OR 0.22 [95% CI 0.05–0.97], *p* = 0.046) and clinical sepsis (OR 0.10 [95% CI 0.02–0.58], *p* = 0.010).

**Conclusions:**

MFR in preterm infants with enterostomy was associated with lower sepsis rates supporting its potential as a safe intervention. Standardized refeeding protocols and prospective studies are warranted to confirm these benefits and to evaluate long-term outcomes after stoma reversal.

**Supplementary Information:**

The online version contains supplementary material available at 10.1186/s40348-026-00227-2.

## Background

Very low birthweight infants (VLBWI) with severe abdominal complications frequently undergo enterostomy as part of a life-saving surgical management. These infants represent a particularly vulnerable population [1] with a high risk of infectious [2], nutritional [3], and metabolic complications [4, 5]. Immaturity of the intestinal barrier, prolonged dependence on parenteral nutrition (PN) [6], and repeated central venous access predispose these infants to systemic inflammation and sepsis, which remain major contributors to morbidity and adverse neurodevelopmental outcomes [7–9].

Although enterostomy allows decompression and prevents further peritoneal contamination, it also diverts the fecal stream and excludes the distal bowel from nutrient and microbiota exposure. Optimizing postoperative enterostomy management is therefore a key clinical priority in neonatal care [10].

Mucous Fistula Refeeding (MFR) — the reinfusion of proximal bowel effluent into the distal mucous fistula — has been proposed as a physiological approach to preserve mucosal integrity, stimulate distal bowel adaption and reduce central line exposure with potential downstream reductions of infection risk and PN-associated cholestasis [11–14]. Although adoption of MFR is increasing, the existing literature remains sparse, particularly with regard to clinical outcomes and associated risks [13]. A recent meta-analysis identified 17 articles statistically relevant, mostly of retrospective, single-center cohort nature and one randomized controlled trial [13]. Findings regarding nutritional outcomes, cholestasis, surgical recovery, and infectious complications are inconsistent, in particular data on sepsis—one of the most clinically relevant and potentially modifiable outcomes in VLBWI. Although several investigations have suggested potential benefits for infection control and nutritional recovery, large multicenter data in VLBWI remain limited. As a result, uncertainty persists regarding the effectiveness and safety of MFR in routine neonatal care.

The primary objective of this study was to evaluate the association between MFR and infectious outcomes, particularly clinical and culture-proven sepsis, in very low birth weight infants undergoing enterostomy. Secondary objectives included assessment of antibiotic exposure, cholestasis parameters, and growth outcomes until stoma reversal.

## Methods

### Ethics

Approval by the local ethics committee for research in human subjects of the University of Lübeck (file number 08–022 and 14–220) and by the local ethics committees of all participating centres has been granted. The GNN was funded by the German Ministry for Education and Research (BMBF-grant-No: 01ER0805 and 01ER1501).

### Objectives and study population

We conducted a multicenter cohort study within the German Neonatal Network (GNN). The primary objective of this study was to evaluate the association between MFR and postoperative infectious morbidity in VLBWI undergoing enterostomy. The primary outcomes of this study were the prevalence of blood culture–proven sepsis and clinical sepsis, defined according to national surveillance criteria, occurring after stoma creation until reanastomosis. Secondary outcomes included recurrent inflammatory activity, defined as at least two elevations of C-reactive protein (CRP) greater than 1 mg/dl separated by a minimum of 14 days and with an intervening decline below 0.5 mg/dl. In addition, the total number and cumulative duration of antibiotic therapies administered between enterostomy creation and stoma reversal were assessed, as well as the number and cumulative duration of central venous catheter (CVC) exposure. Cholestasis parameters—total bilirubin, direct bilirubin, and gamma-glutamyl transferase (GGT)—were evaluated at the time of stoma reversal and at the estimated due date. Growth parameters, including weight, length, and head circumference, were recorded at predefined time points: enterostomy creation, stoma reversal, 36 weeks postmenstrual age, estimated date of delivery, and discharge. Finally, the reoperation rate following stoma reversal was analyzed. All outcomes were assessed from the time of enterostomy formation until surgical reanastomosis unless otherwise specified.

The GNN is a multicenter, population-based cohort enrolling VLBWI throughout Germany with standardized data collection and annual site monitoring (www.vlbw.de). GNN centers were contacted for willingness to participate in this study and to provide information on postoperative enterostomy management. To achieve comparable group sizes, participating sites were selected to ensure representation of both centers routinely performing MFR and centers not applying MFR in their clinical practice. For the present analysis, data from infants of five GNN sites were included. Of these centers, all VLBWI < 1500 g birth weight, who were born between January 1, 2009, and December 31, 2022 and underwent enterostomy due to abdominal complications were screened for eligibility. Other inclusion criteria included complete documentation of the abdominal situation, nutrition and methodology of MFR, and survival until discharge.

### Collection of laboratory data

Laboratory values originated from respective local clinical laboratories of the participating centers. Cholestasis parameters such as bilirubin, direct bilirubin, and gamma-glutamyl transferase were recorded on the day of enterostomy, if available. Their absence was not considered an exclusion criterion, as cholestasis was not the primary outcome of this study. CRP levels were determined from serum or plasma samples obtained at the discretion of the attending physicians for clinical indications, such as suspected infection or monitoring of a confirmed infection. Laboratory values were obtained during routine clinical care at the respective participating centers. For study purposes, these values were retrospectively extracted from local hospital laboratory information systems and transferred into the GNN database for analysis. Measurements were performed according to clinical indication rather than according to a predefined study-specific sampling schedule.

### Definitions

Sepsis after stoma creation was defined according to the criteria of the national infection surveillance system [15]. Clinical sepsis was defined as sepsis with at least two clinical signs (temperature > 38 °C or < 36.5 °C, tachycardia > 200/min, new onset or increased frequency of bradycardias or apneas, hyperglycemia > 140 mg/dl, base excess < − 10 mval/l, pale/grey skin color, increased oxygen requirements) or one clinical and one laboratory sign (C-reactive protein (CRP) > 1 mg/dl), but no proof of causative agent in the blood culture. Blood-culture proven sepsis was defined as clinical sepsis with proof of causative agent in the blood culture. As we focused on postsurgical inflammatory activity, CRP values obtained on postoperative day three were included as an early standardized marker of inflammation. However, sepsis definitions were not restricted to this timepoint and were based on clinical and microbiological criteria throughout the entire period from enterostomy creation until reanastomosis.

Recurrent CRP elevation was defined as ≥ 2 CRP values > 1 mg/dl, separated by at least 14 days, with an intervening decline below 0.5 mg/dl to ensure that new peaks were distinguished from persistently elevated levels.

The duration of antibiotic therapy was defined as the cumulative number of days with antibiotics from enterostomy formation until reanastomosis. Growth parameters (weight, length, and head circumference) were documented in grams and centimeters at defined timepoints: at enterostomy creation, at reanastomosis, at 36 weeks postmenstrual age, at estimated date of delivery, and on the day of discharge.

### Statistical analysis

Baseline characteristics are presented as median with interquartile range (IQR) for continuous variables and as number with percentages with 95% confidence intervals (CIs) for categorical variables. Group comparisons were performed using the Mann–Whitney U test for continuous variables and the χ^2^ test for categorical variables. A two-sided p-value < 0.05 was considered statistically significant for individual comparisons.

Multivariable logistic regression was used to evaluate associations between MFR and infection outcomes, adjusting a priori for gestational age, birthweight, number of central venous catheters, probiotic use, and total antibiotic exposure. Treating center was included as a fixed-effect variable to account for potential inter-center variability in sepsis incidence. Model diagnostics (residuals, collinearity) were assessed to confirm adequacy.

Statistical analyses were performed with SPSS software (IBM SPSS Statistics for Windows, Version 29.0. Munich, Germany).

## Results

### Study population

**Fig. 1:**
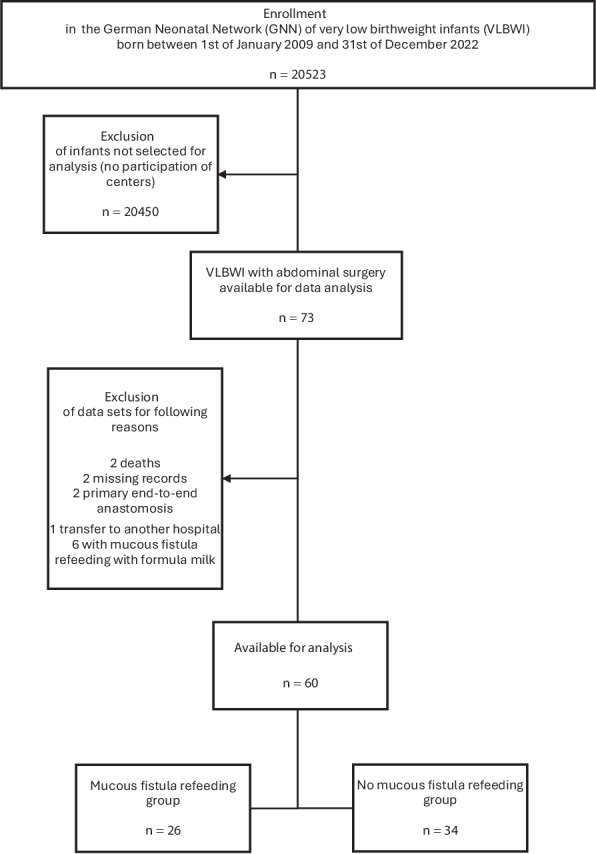
Flowchart of in- and exclusion of participants

Between January 2009 and December 2022, a total of 20,523 VLBWI were enrolled in the German Neonatal Network (GNN). Five centers participated in our retrospective analysis. Here, 73 infants with abdominal surgery were identified for analysis. Seven were excluded due to death (*n* = 2), missing medical records (*n* = 2), primary end-to-end anastomosis (*n* = 2), or transfer to another hospital (*n* = 1). The remaining 66 infants formed the study cohort, of whom 60 were included in the final analysis after exclusion of six cases with formula refeeding. Of these, 26 infants underwent MFR and 34 infants did not (Fig. 1).

Mucous fistula refeeding (MFR) was performed according to local center protocols, which were comparable in principle but not formally standardized across all sites. In general, proximal stoma effluent was collected under sterile conditions and reinfused into the distal mucous fistula shortly after collection. Refeeding was performed intermittently (*n* = 22, 84.6%) rather than continuously (*n* = 4, 15.4%). Effluent was typically administered several times per day in small aliquots, depending on the volume of proximal output and the infant’s clinical condition. Administration was performed manually using sterile syringes via a soft feeding catheter inserted into the mucous fistula. Infusion pumps were not used. The volume reinfused corresponded to the collected proximal output, adjusted when necessary, according to tolerance (e.g., abdominal distension, increased gastric residuals, or leakage at the fistula site). No standardized protocol regarding exact frequency, aliquot size, or duration of each administration was mandated within the GNN framework. Thus, minor inter-center variability cannot be excluded.

### Cohort data

Baseline clinical characteristics were comparable between infants with and without MFR (see Table 1).

**Table 1:** Baseline cohort characteristics

Variable	Mucous fistula refeeding (*n* = 26)	No Mucous fistula refeeding (*n* = 34)
Gestational age (weeks) ^#^	25.4 (25.3 [24–26.1])	25.4 (24.5 [23.9–26.1])
Birth weight (gramm) ^#^	724 (660 [595–835]	726 (720 [530–860])
SGA (< 10th percenile)	7 (19.4 [9.1–34.4])	9 (31.0 [16.6–49.0])
Female	14 (53.8 [35.1–71.8])	16 (47.1 [31.1–63.5])
multiple gestation	8 (30.8 [15.8–49.8])	13 (38.2 [23.4–55.0])
spontaneous delivery	4 (15.4 [5.4–32.5])	10 (29.4 [16.2–45.9])
C-section	18 (69.2 [50.2–84.2])	16 (47.1 [31.1–63.5])
emergency C-section	4 (15.4 [5.4–32.5])	8 (23.5 [11.8–39.5])
APGAR at 5 min	7 (7–8)	8 (6–8)
APGAR at 10 min	8 (8–9)	9 (8–9)
APGAR at 5 min < 7	5 (14.3 [5.7–28.5])	9 (31.0 [16.6–49.0])
Umbilical arteries pH	7.34 (7.26–7.37)	7.38 (7.34–7.31)
Maximum Fraction of inspired Oxygen within first 12 h of life	48 (33–80)	48 (35–90)
Vasopressor use within first 24 h of life	12 (35.5 [20.9–52.0])	8 (29.6 [15.1–48.2])
Mechanical ventilation within first 7 days of life	31 (86.1 [72.2–94.5])	28 (96.6 [85.0–99.6])
Total days of mechanical ventilation	19.5 (7.5–40.0)	25.0 (13.0–38.0)
antenatal steroids	23 (88.5 [72.3–96.6])	31 (91.2 [78.3–97.5])
completed antenatal steroids	7 (53.8 [28.3–77.9])	7 (58.3 [31.2–82.0])
antenatal antibiotics	15 (57.7 [38.7–75.0])	20 (60.6 [43.6–75.8])
ICH (all grades)	7 (26.9[12.9–45.7])	14 (41.2 [25.9–57.9])
PVL	3 (11.5 [3.4–27.7])	2 (5.9[1.2–17.6])
NEC requiring surgery	20 (55.6 [39.4–70.8])	16 (55.2 [37.3–72.1])
SIP requiring surgery	14 (38.9 [24.3–55.2])	12 (41.4 [25.0–59.4])
Postnatal age at enterostomy formation [days]	15.0 (6–30)	10.0 (8–21)

Baseline cohort characteristics stratified by mucous fistula refeeding practice (MFR). Categorical variables are given as n (%) with corresponding 95% confidence interval (CI); continuous variables as median (IQR) if appropriate (#)

Abbreviations: *SGA*  Small for gestational age, *C-section*  Caesarean section, *ICH*  Intracerebral hemorrhage, *PVL*  Periventricular leukomalacia, *NEC*  Necrotizing enterocolitis, *SIP*  Spontaneous intestinal perforation

The median gestational age and birth weight were similar in both groups with slightly higher proportion of females among MFR infants. Spontaneous delivery was less frequent among MFR infants (15.4% [6.8–28.1] vs. 29.4% [17.5–43.9]), while emergency caesarean sections were performed in 15.4% [6.8–28.1] in the MFR infants and 23.5% [12.3–37.8] in no MFR infants. Exposure to antenatal steroids and maternal antibiotic treatment prior to delivery showed comparable frequencies. Main neurological outcome characteristics were also similar in both groups.

### Univariate analyses

In univariate comparisons, VLBWI with fecal transfer showed significantly lower frequencies of blood-culture–proven sepsis (11.5% [95% CI 3.4–27.7] vs. 36.4% [21.6–53.4], *p* = 0.030) and clinical sepsis (50.0% [31.6–68.4] vs. 78.8% [62.8–90.0], *p* = 0.020) compared to infants without MFR (see Table 2).

**Table 2:** Univariate analysis of clinical outcomes and laboratory parameters stratified by mucous fistula refeeding

Variable	Mucous fistula refeeding (*n* = 26)	No mucous fistula refeeding (*n* = 34)	*P*-Value
Clinical sepsis	13 (50.0 [31.6–68.4])	26 (78.8 [62.8–90.0])	0.020
Blood culture–proven sepsis	3 (11.5 [3.4–27.7])	12 (36.4 [21.6–53.4])	0.030
Any Infection and germ detection at CVC	12 (38.7 [23.3–56.2])	7 (46.7 [23.9–70.6])	0.607
Reoperation after stoma reversal	3 (12.5 [3.6–29.7])	10 (31.3 [17.3–48.4])	0.100
Recurrent CRP Elevation > 1 mg/dl	12 (46.2 [28.2–64.9])	25 (75.8 [59.4–87.8])	0.020
Total number of antibiotic therapies	7.00 (4.00–8.00)	7.65 (5.00–10.00)	0.024
Total duration of antibiotic therapy	80.73 (41.00–106.00)	91.44 (68.00–122.00)	0.071
Number of CVCs	3 [1–3]	3 (2–4)	0.024
Total bilirubin at stoma reversal day (mg/dl) ^Ω^	2.08 (0.2–3.06)	2.29 (0.4–4.6)	0.769
Direct bilirubin at stoma reversal day (mg/dl) ^Ω^	1.19 (0.00–2.40)	1.41 (0.00–2.55)	0.608
GGT at stoma reversal day (mg/dl) ^Ω^	107 (47–87)	145 (78–146)	0.069
Total bilirubin at estimated due date (mg/dl) ^Φ^	3.18 (0.50–6.340)	3.82 (0.60–4.30)	0.605
Direct bilirubin at estimated due date (mg/dl) ^Φ^	2.71 (0.22–4.40)	3.10 (0.00–5.30)	0.838
GGT at estimated due date (mg/dl) ^Φ^	244 (86–381)	173 (76–304)	0.423
Weight at 36 weeks GA (g)	1747 (1530–2000)	1863 (1620–2230)	0.382
Length at 36 weeks GA (cm)	41.6 (39.0–43.0)	41.6 (39.0–43.0)	0.593
Head circumference at 36 weeks GA (cm)	28.8 (27.2–31.0)	29.4 (28.0–31.0)	0.305
Weight at stoma reversal day (g)	3212 (2140–3576)	2679 (1983–2913)	0.150
Length at stoma reversal day (cm)	49.3 (43.5–57.0)	46.4 (42.0–43.0)	0.165
Head circumference at stoma reversal day (cm)	33.3 (30.0–36.4)	31.5 (30.0–36.4)	0.192
Weight at estimated due date (g)	2374 (1965–2400)	2539 (2275–2795)	0.158
Length at estimated due date (cm)	46.3 (43.0–48.5)	44.6 (42.0–47.5)	0.343
Head circumference at estimated due day (cm)	31.9 (30.0–34.0)	32.0 (31.0–33.0)	0.677
Discharge with nasogastric tube	2 (8.0 [1.7–23.3])	6 (19.4 [8.5–35.6])	0.227
Duraction of central line catheter exposure [days]	57.5 (29.0–91.5)	41 (24.0–80.0)	0.553

Categorical variables are presented as n (% [95% CI]); continuous variables as median (IQR).

Abbreviations: *MFR* Mucous fistula refeeding, *CVC* Central venous catheter, *GA* Gestational age, *CRP* C-reactive protein, *GGT* Gamma-glutamyltransferase, *EDD* Estimated due date

^**Ω**^ *n* = 32 cases missing

^**Φ**^ *n* = 29 cases missing

Recurrent CRP elevations > 1 mg/dl occurred less frequently in the MFR group (46.2% [28.2–64.9] vs. 75.8% [59.4–87.8], *p* = 0.020). MFR infants received fewer antibiotic courses (7.0 [4.0–8.0] vs 7.7 [5.0–10.0]; *p* = 0.024), with a non-significant trend toward shorter antibiotic duration (80.7 [41.0–106.0] vs 91.4 [68.0–122.0] days; *p* = 0.071) among MFR infants.

No significant differences were observed in GGT values and total or direct bilirubin at either stoma reversal or estimated due date. Growth parameters (weight, length, head circumference) were also comparable at all timepoints. Although the reoperation rate after stoma reversal was lower in MFR infants ((12.5% [95% CI 4.2–26.8] vs. 31.3% [95% CI 18.7–46.3]) this did not reach statistical significance (*p* = 0.100).

Overall, univariate analyses indicate that MFR is associated with a substantially lower infection burden, without evidence of increased surgical or metabolic complications.

### Adjusted analyses

To account for potential confounding, multivariable logistic regression was performed. Scatterplot and residual diagnostics confirmed model adequacy and absence of multicollinearity. The overall model fit was acceptable, with Nagelkerke R^2^ values of 0.155 for blood culture–proven sepsis and 0.347 for clinical sepsis, indicating a moderate explanatory power. Here, MFR remained independently associated with reduced odds of blood-culture–proven sepsis (OR 0.22 [95% CI 0.05–0.97], *p* = 0.046) and clinical sepsis (OR 0.10 [95% CI 0.02–0.58], *p* = 0.010) (see Table 3).

**Table 3:** Multivariable logistic regression analyses of variables associated with blood culture–proven sepsis and clinical sepsis

Variables	Bloodculture proven sepsis		Clinical sepsis	
	**OR (95%CI)**	***p*-value**	**OR (95%CI)**	***p*-value**
MFR	0.22 (0.05–0.97.05.97)	0.046	0.10 (0.02–0.58)	0.010
Gestational age	0.89 (0.55–1.46)	0.646	0.56 (0.32–0.99	0.045
Birth weight	1.0 (0.99–1.00.99.00	0.945	1.00 (0.99–1.00)	0.443
Number of CVCs after stoma creation	0.77 (0.44–1.35	0.363	0.77 (0.43–1.37)	0.370
Probiotic use during MFR	0.90 (0.20–4.09	0.889	0.88 (0.16–4.72)	0.880
Total number of antibiotic therapies	0.91 (0.73–1.13)	0.378	1.18 (0.93–1.49)	0.176
Participating clinic	0.99 (0.94–1.1.94.1)	0.853	1.04 (0.97–1.12.97.12)	0.227
Nagelkerke R.^2^	0.155		0.347	

Models adjusted for gestational age, birth weight, number of central venous catheters (CVC), probiotic use, and total number of antibiotic therapies

Abbreviations: *CI*  Confidence interval, *OR*  Odds ratio, *MFR*  Mucous fistula refeeding

## Discussion

This multicenter retrospective analysis within the GNN demonstrates that MFR is associated with a substantially lower incidence of both culture-proven and clinical sepsis in preterm infants with enterostomy. The effect remained significant after adjustment for known covariates, suggesting that the reduction in infectious complications is not explained by baseline differences but reflects an independent benefit of refeeding.

Previous studies evaluating infectious outcomes following mucous fistula refeeding have yielded heterogeneous results. Septic complications in refed infants have been reported in some cohorts; however, these observations were derived from small, single-center studies with limited statistical power and heterogeneous clinical practices [16, 17]. In contrast, several investigations found no significant differences in infection rates between refed and non-refed infants, suggesting at least a neutral effect of MFR on sepsis risk [12, 14, 18].

The substantially lower sepsis rates observed in the present multicenter cohort are consistent with these latter findings and extend them by demonstrating a protective association in a population-based setting with adjustment for relevant confounders. It remains possible that variability in sepsis incidence across studies reflects differences in local refeeding protocols, timing of initiation, handling procedures, and overall postoperative management rather than the refeeding strategy itself.

Mechanistic evidence supports the observed association between mucous fistula refeeding and reduced infectious morbidity. Abdominal surgery in preterm infants has been shown to disrupt intestinal bacterial diversity [19] and promote enteral deprivation of the distal bowel, thereby increasing susceptibility to infection through impaired barrier function and potential transluminal passage of pathogens. In contrast, histopathological findings demonstraded increased mucosal thickness and preservation of villous architecture in the distal ileum at the time of stoma closure, when infants received MFR. Furthermore, an increased lymphoid follicle formation within the mucosa in these infants suggests that MFR may mitigate infection risk by maintaining distal bowel integrity and supporting mucosal immune defense [20].

Beyond local intestinal effects, reduced exposure to PN has been proposed as an additional mechanism contributing to lower sepsis rates in infants receiving MFR, potentially through decreased reliance on central venous access [21]. In our cohort, infants who developed infections tended to have longer cumulative exposure to central venous catheters. However, this association did not reach statistical significance, indicating that factors beyond central line duration are more likely to contribute to the observed reduction in sepsis.

The reoperation rate after stoma reversal was numerically lower in the MFR group. Previous work described improved bowel conditioning before reanastomosis among infants receiving refeeding and smoother postoperative recovery in refed neonates [21–24]. Although our findings did not reach statistical significance, the trend suggests that maintaining distal bowel activity may support structural readiness for surgical reconstruction.

Cholestasis parameters showed no significant differences between the groups. This contrasts with studies reporting improved bile flow and reduced parenteral nutrition–associated cholestasis in refed infants [14, 24]. In our study, however, cholestasis markers were not consistently available, limiting the interpretability of these results.

Somatic growth, evaluated through weight, length, and head circumference, was comparable between infants with and without refeeding at all predefined timepoints. While earlier studies described improved growth parameters in refed infants [17, 25], our cohort did not demonstrate clear growth advantages. However, the comparable growth patterns across both groups indicate that MFR does not negatively affect metabolic development in this vulnerable population.

Several limitations of this study should be acknowledged. First, the retrospective design is inherently susceptible to selection bias, as the decision to implement mucous fistula refeeding was based on clinical judgment and center-specific protocols rather than random allocation. Inter-center variability in postoperative management, including antibiotic exposure, probiotic use, and enteral feeding strategies, may therefore have influenced observed outcomes. Nevertheless, the consistent direction and magnitude of associations across both univariate and multivariable analyses support the internal robustness of our findings.

Second, the availability of laboratory data was incomplete, particularly with regard to cholestasis markers, limiting the ability to fully assess metabolic and hepatobiliary effects of MFR. In addition, several clinically relevant outcomes could not be reliably evaluated. Time to achievement of full enteral feeding, and post-discharge outcomes were not systematically captured in the database. These parameters were excluded from analysis due to incomplete or inconsistent documentation across centers, which could have introduced substantial misclassification bias. Finally, although this represents one of the largest multicenter cohorts in very low birth weight infants, the overall sample size remains limited, restricting statistical power for subgroup and interaction analyses. As a result, smaller effects, particularly for secondary outcomes, may have gone undetected.

Beyond potential benefits, practical challenges of MFR should be considered. The procedure requires meticulous handling of proximal effluent, strict hygienic precautions, and trained personnel to avoid contamination during collection and reinfusion. Repeated catheterization of the mucous fistula may be associated with local irritation, leakage, or mechanical trauma, particularly in extremely preterm infants with fragile tissues. In addition, variability in stoma output volume and consistency may complicate standardized administration. From a logistical perspective, MFR increases nursing workload and requires clear protocols to ensure safe and consistent application. Concerns have also been raised regarding the theoretical risk of ascending infection or bacterial translocation, although current clinical data, including our findings, do not indicate an increased incidence of bloodstream infections. Careful patient selection, standardized procedures, and close clinical monitoring therefore remain essential when implementing MFR in routine practice.

## Conclusions

From a clinical perspective, the consistent reduction in sepsis rates observed in our cohort suggests that MFR may represent a valuable infection-preventive strategy in infants with enterostomy. Even moderate reductions in bloodstream infections can have far-reaching implications, including shorter antibiotic exposure, reduced central line use, and improved overall stability. The physiological rationale of restoring luminal continuity underlines the potential of MFR to mitigate systemic inflammation in preterm infants. Growth neutrality and the absence of procedure-related complications strengthen the conclusion that MFR is safe when infants are clinically stable and protocols are standardized. MFR therefore appears to be a safe and feasible adjunct in the postoperative care of preterm infants with enterostomy. Prospective multicenter trials are needed to further define optimal timing, procedural standardization and underlying mechanisms, and to determine the long-term clinical impact in VLBWI [26].

## Supplementary Information

Supplementary Material 1.

## Data Availability

The datasets used and analysed during the current study are available from the corresponding author on reasonable request.

## Abbreviations

BMBF: Bundesministerium für Bildung und Forschung (German Federal Ministry of Education and Research)
BPD: Bronchopulmonary Dysplasia
CI: Confidence Interval
C-Section: Cesarean section
CRP: C-Reactive Protein
CVC: Central venous catheter
GA: Gestational Age
GGT: Gamma-Glutamyl Transferase
GNN: German Neonatal Network
ICH: Intracerebral Hemorrhage
IQR: Interquartile Range
MFR: Mucous Fistula Refeeding
NEC: Necrotizing Enterocolitis
OR: Odds Ratio
PN: Parenteral Nutrition
PVL: Periventricular Leukomalacia
SIP: Spontaneous Intestinal Perforation
VLBWI: Very Low Birth Weight Infant(s)
