# Supplementary material for: Sepsis profile among preterm infants with enterostomy and fecal transfer: a multicenter retrospective cohort study
Source: Mol Cell Pediatr. 2026 Apr 10;13:15. doi: 10.1186/s40348-026-00227-2 (PMC13069041; doi:10.1186/s40348-026-00227-2)
Supplement: Supplementary file 1 — Supplementary Material 1. [file 40348_2026_227_MOESM1_ESM.docx]

**Supplementary Material**

**Supplementary table 1: Pathogens in blood culture-positive infections**

| **Pathogen** | **Mucous fistula refeeding**  **(n = 3)** | **No Mucous fistula refeeding**  **(n = 12)** |
| --- | --- | --- |
| **Coagulase negative staphylococcus** | 2 (50.0 [12.3-87.7]) | 9 (64.3 [38.5-84.9]) |
| **Enterobacter cloacae** | n.a. | 1 (7.1 [0.8-28.8]) |
| **Enterobacter aerogenes** | n.a. | 1 (7.1 [0.8-28.8]) |
| **Enterococcus faecalis** | n.a. | 1 (7.1 [0.8-28.8]) |
| **Candida albicans** | 1 (25.0 [2.8-71.6]) | n.a. |
| **Bifidobacterium** | n.a. | 1 (7.1 [0.8-28.8]) |
| **Streptococcus agalactiae** | n.a. | 1 (7.1 [0.8-28.8]) |
| **Streptococcus bovis** | 1 (25.0 [2.8-71.6]) | n.a. |

Detected pathogens in blood culture-positive infections stratified by mucous fistula refeeding practice (MFR). Categorical variables are given as n (%) with corresponding 95 % confidence interval (CI); In total, 22 pathogens were detected; double positive blood-cultures explain the higher number of bacterial detections as evidence of infections. Abbreviations: n.a. = not applicable
